# Mönckeberg sclerosis in patients with thyroid papillary carcinoma

**DOI:** 10.1530/EO-23-0047

**Published:** 2024-05-02

**Authors:** Vasiliki Venetsanaki, Eleana Zisimopoulou, Chrysanthi Zouli, Maria Boudina, Konstantinos Gkiouras, Persefoni Xirou, Aimilia Fotiadou, Mariana Stamati, Elpiniki Argyropoulou, Alexandra Chrisoulidou

**Affiliations:** 1 Department of Endocrinology, Theagenio Hospital, Thessaloniki, Greece; 2 Department of Pathology, Theagenio Hospital, Thessaloniki, Greece

**Keywords:** Mönckeberg medial sclerosis, papillary thyroid cancer, thyroid

## Abstract

**Background:**

Mönckeberg sclerosis is a form of calcification of the tunica media of small and medium size arteries. It occurs more often in the peripheral arteries of the lower limbs and it has been associated with diabetes and renal disease. Although there are a few reports of Mönckeberg sclerosis in thyroid vessels, there are no data regarding its significance in thyroid disease.

**Objective:**

The aim was to investigate the possible prognostic value of Mönckeberg sclerosis in thyroid vessels of patients with diagnosed thyroid cancer.

**Methods:**

We retrospectively studied patients with papillary thyroid cancer treated at the Theagenio Hospital of Thessaloniki from 2005 to 2021. The patients were divided into two groups based on the presence, or absence, of histopathological findings of Mönckeberg sclerosis in the thyroid vessels along with papillary thyroid cancer. Patient characteristics, histopathological details, personal history of thyroid disease, and metabolic parameters were compared between the two groups.

**Results:**

Thirty-three patients with papillary thyroid carcinoma and Mönckeberg sclerosis were identified and matched to 33 controls with papillary thyroid cancer, without evidence of Mönckeberg sclerosis. The metabolic profile of patients with Mönckeberg sclerosis was not significantly different from those who did not have Mönckeberg sclerosis. Moreover, the comparison between the two groups did not reveal any remarkable differences in terms of the aggressiveness of the disease.

**Conclusion:**

The presence of Mönckeberg sclerosis does not seem to impact on histological characteristics of patients with papillary thyroid cancer.

## Introduction

**Figure 1:**
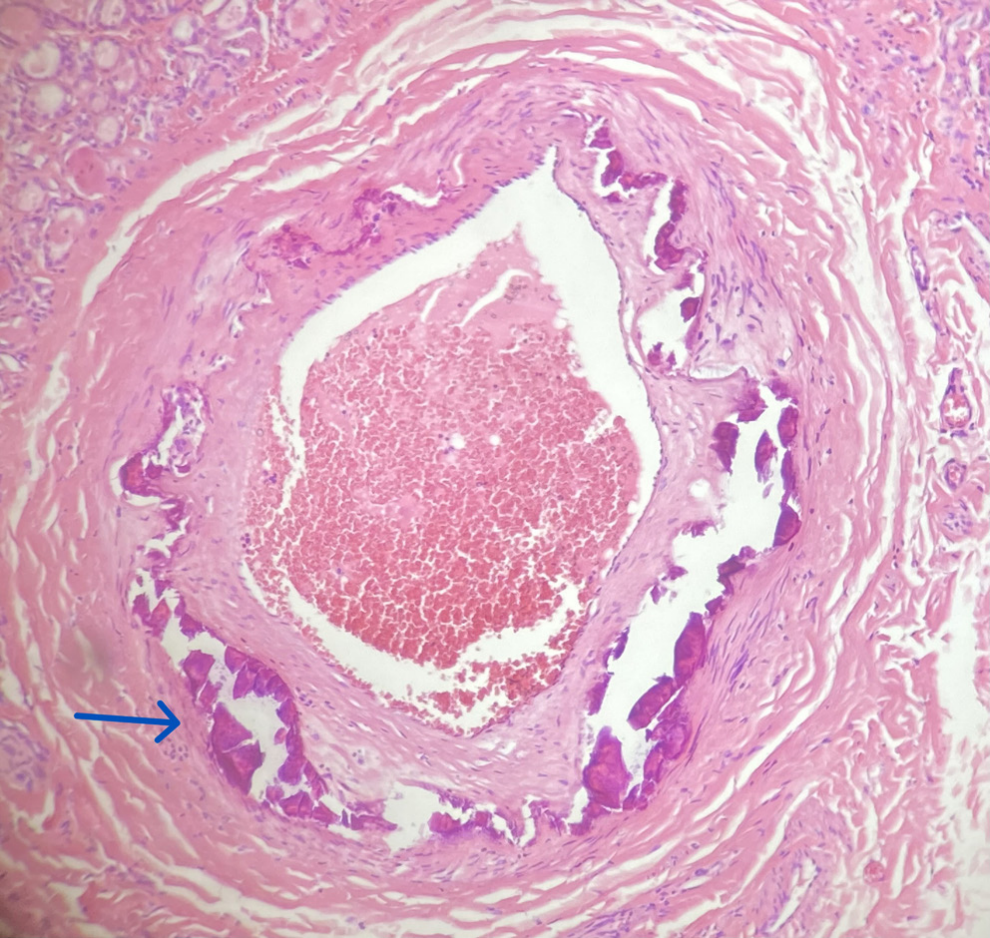
Calcium deposits (blue arrow) in the media of a muscular artery.

Mönckeberg medial sclerosis (MS) is the calcification of the tunica media of muscular arteries (Fig. 1). Unlike intima calcifications, medial calcifications do not cause luminal obstruction. However, over time, the decrease in elasticity and compliance of the vessel may lead to atherosclerosis and has been associated with increased cardiovascular risk (Niskanen *et al*. 1994, London *et al*. 2003, Rocha-Singh *et al*. 2014). Four stages of MS severity have been described: the first stage includes only irregular deposits embedded within the media, the second stage involves up to three quadrants of the vessel, and the third stage involves the entire circumference of the vessel. In stage four, foci of bone formation can be found inside the arterial media (osseous metaplasia) (Lanzer *et al*. 2014).

Recognized risk factors for MS include advanced age, type II diabetes mellitus, and renal disease. Disorders of calcium metabolism such as hyperparathyroidism, vitamin D disorders, osteoporosis, vitamin K disorders, and rheumatoid arthritis have also been associated with MS (Lanzer *et al*. 2014). It can be visible on plain X-rays with a typical railroad track-type pattern. Mönckeberg sclerosis is most commonly encountered in small and medium-sized arteries of the extremities. However, MS has also been reported in the coronary, temporal, uterine, ovarian, and mammary arteries. The presence of MS in thyroid vessels has been previously described as a histopathologic finding (Micheletti *et al*. 2008, Dastamani *et al*. 2010). However, MS in benign and malignant thyroid specimens has not been studied so far. Early studies discussed cancers with a low rate of growth in patients with calcified atherosclerosis. Nevertheless, neither thyroid cancer nor Mönckeberg sclerosis has been studied over the years in this context, and relevant literature is scanty. The aim of our study was to examine if MS contributes to some tumoral characteristics. The hypothesis we had was that in patients with MS, invasive characteristics were seen less often than in patients without MS.

## Materials and methods

### Study design and subjects

Thyroidectomy records from 2005 to 2021 were retrospectively evaluated. Thirty-three patient pathology reports that specify the diagnosis of a papillary thyroid carcinoma and the presence of MS in thyroid blood vessels were included as cases. We identified thirty-three controls from the same cohort matched for age, gender, and primary diagnosis (PTC). All cases and controls were reviewed by the same expert pathologist in our institute. MS was specifically looked for in all thyroid specimens. Data regarding patient information, including age at diagnosis, body mass index, smoking status, history of thyroid disease, dyslipidemia, diabetes mellitus, and hypertension, and pathology characteristics, including papillary thyroid subtype, multifocality, disease location, disease extension, and total time of follow-up, were collected from patient records (Table 1). All patients signed an informed consent form.

**Table 1:** Characteristics of cases and controls.

Characteristic	*n*^a^	Total sample	*n*	Cases	*n*	Controls	*P*
		Statistic^b^		Statistic^b^		Statistic^b^	
Age at diagnosis, years	66	57.3 ± 12.4	33	56.8 ± 11.8	33	57.8 ± 12.4	0.741^c^
Follow-up, years	50	4.5 (1–8.3)	25	4.0 (1.0–7.0)	25	8.0 (2.0–11.0)	0.109^d^
BMI, kg/m^2^	47	28.3 (24.8–33.3)	23	27.7 (24.0–34.1)	24	29.2 (25.1–31.9)	1.000^d^
Smokers	46	32.6%	24	29.2%	22	36.4%	0.603^e^
Tumor size, mm	63	6.0 (3.0–13.0)	30	5.0 (3.8–12.8)	33	7.0 (3.0–13.0)	0.783^d^
CCr	45	118.2 (82.4–146.8)	24	119.8 (84.5–147.2)	21	118.2 (78.2–144.4)	0.891^d^

^a^Refers to sample with available data;^ b^Data are being presented as means ± s.d., medians (first and third quartiles), or percentages; ^c^Based on the Student’s *t*-test; ^d^Based on the Mann–Whitney *U*-test; ^e^Based on the chi-square test.

BMI, body mass index; CCr, creatinine clearance, Cockcroft–Gault equation.

### Statistical analysis

The normality of continuous data was assessed via the Shapiro–Wilk test and visual examination of the Q–Q plots. Throughout the text, continuous variables are being presented as means ± s.d. for normally distributed variables or as medians along with their first and third quartiles for non-normally distributed variables. Categorical data are being presented as percentages. The Student’s *t*-test was utilized to assess differences between cases and controls when data were normally distributed, and the Mann–Whitney test was used for non-normally distributed continuous data. The chi-square test was used for categorical data. To assess the impact of having MS on the study’s variables, a series of regression analyses were used, with the case–control status being the independent variable and the study’s variables being the dependent variables. Binary logistic regression was used for binary dependent variables, multinomial logistic regression for the categorical dependent variables, and linear regression for the continuous variable of maximum tumor size. For the linear regression, assumptions of normality and homoscedasticity were assessed on the residuals, and since they were found to be violated, the maximum tumor size was transformed via the natural logarithm, and subsequently, the analysis was rerun. All analyses were performed on the SPSS version 25.0 (SPSS, Chicago, IL, USA), and the level of significance was set at *α* = 0.05 in the analyses comparing the sample’s characteristics and at *α* = 0.003125 in the logistic regressions after applying the Bonferroni correction.

## Results

### General characteristics

Thirty-three patients with papillary thyroid carcinoma and Mönckeberg sclerosis were identified. All patients were females. The cases were matched for age, gender, and PTC to 33 controls. Overall, 66 female patients participated in the study with a mean age of 57.3 years at the time of their diagnosis (Table 1). Smoking status and BMI did not vary between groups; 32% of our population were smokers, and the median BMI was high in both groups. Maximal dimensions of the papillary thyroid carcinoma were lower in cases than controls, although their difference was not statistically significant (*P* = 0.783).

### Impact of case–control status on study variables

Table 2 illustrates the effect of MS on a number of pathologies and comorbidities.

**Table 2:** Logistic regressions regarding the impact of case–control status (independent variable) on the study variables.

Variable	Cases, *n*	Controls, *n*	OR (95% CI)	*P*
Pathology				
Classic PTC	19	16	Ref.	
PTC-FV/encapsulated PTC-FV	14	17	0.69 (0.26–1.83)	0.460
Multifocal				
No	15	20	Ref.	
Yes	17	13	1.74 (0.65–4.67)	0.268
Invasion of parenchyma				
No	20	23	Ref.	
Yes	12	9	1.53 (0.54–4.39)	0.426
Thyroid capsule invasion				
No	24	27	Ref.	
Yes	8	6	1.50 (0.46–4.94)	0.505
Extrathyroidal invasion				
No	24	26	Ref.	
Yes	8	6	1.44 (0.44–4.77)	0.546
Lymph node disease				
No	11	12	Ref.	
Yes	1	3	0.30 (0.03–3.15)	0.316
No dissection	20	18	0.83 (0.29–2.33)	0.716
Multinodular goiter				
No	3	8	Ref.	
Yes	29	25	3.09 (0.74–12.94)	0.122
Hyperthyroidism (Graves’ disease or other)				
No	27	21	Ref.	
Yes	4	5	0.62 (0.15–2.61)	0.516
Autoimmune thyroiditis				
No	7	12	Ref.	
Yes	10	8	2.14 (0.57–7.99)	0.257
Thyroid ultrasound calcifications present				
No	9	10	Ref.	
Yes	12	9	1.48 (0.43–5.16)	0.537
FNA result				
Benign	12	9	Ref.	
Malignancy	6	6	0.75 (0.18–3.12)	0.692
Non-diagnostic	1	3	0.25 (0.02–2.82)	0.262
Dyslipidemia				
No	4	10	Ref.	
Yes	21	15	3.50 (0.92–13.31)	0.066
Diabetes mellitus				
No	20	19	Ref.	
Yes	5	6	0.79 (0.21–3.03)	0.733
Hypertension				
No	10	13	Ref.	
Yes	14	12	1.52 (0.49–4.69)	0.470

FNA, fine needle aspiration; OR, odds ratio; PTC-FV, papillary thyroid carcinoma follicular variant; Ref., reference category.

The presence of Mönckeberg MS was associated with a 31% decrease in the odds of having the follicular variant of papillary thyroid carcinoma (OR = 0.69, 95% CI: 0.26–1.83, *P* = 0.460) and was also associated with a 74% increase in the odds of having multifocal cancer (OR = 1.74, 95% CI: 0.65–4.67, *P* = 0.268), but these associations were not significant.

Similarly, the presence of MS was associated with a nonsignificant 53% increase in the odds of invasion of the thyroid parenchyma (OR = 1.53, 95% CI: 0.54–4.39, *P* = 0.426), a 50% increase in the odds of thyroid capsule invasion (OR = 1.50, 95% CI: 0.46–4.94, *P* = 0.505), and a nonsignificant 44% increase in the odds of having extrathyroidal invasion (OR = 1.44, 95% CI: 0.44–4.77, *P* = 0.546). Furthermore, MS was associated with a decreased chance of lymph node disease (OR = 0.30, 95% CI: 0.03–3.15, *P* = 0.316) and lymph node dissection (OR = 0.83, 95% CI: 0.29–2.33, *P* = 0.716); however, these associations did not reach statistical significance.

Regarding other common comorbidities, the odds of having dyslipidemia and hypertension were non-significantly increased (OR = 3.50, 95% CI: 0.92–13.31, *P* = 0.066; OR = 1.52, 95% CI: 0.49–4.69, *P* = 0.470, respectively), while they were non-significantly reduced for having type II diabetes mellitus (OR = 0.79, 95% CI: 0.21–3.03, *P* = 0.733). The presence of MS was associated with a 48% increase in the odds of identifying calcifications in a preoperative thyroid ultrasound (OR = 1.48, 95% CI: 0.43– 5.16); however, these results were also not significant (*P* = 0.537). Cases with MS had nonsignificant reduced chances of having an FNA indicating malignancy or a non-diagnostic result (OR = 0.75, 95% CI: 0.18–3.12, *P* = 0.692; OR = 0.25, 95% CI: 0.02–2.82, *P* = 0.262, respectively).

Based on linear regression analysis with the maximum tumor size as a transformed variable, having Mönckeberg sclerosis was associated with a nonsignificant reduced tumor size (*β* = −0.98, 95% CI = −0.58 to 0.39, *P* = 0.687). In addition, it should be noted that these results remained unchanged in terms of their significance when only the original, non-transformed variable of tumor size was inserted in the analysis (*β* = −1.76, 95% CI= −5.87 to 2.5, *P* = 0.394).

## Discussion

To our knowledge, this is the first study that investigates multiple factors associated with the presence of Mönckeberg sclerosis in patients with papillary thyroid carcinoma. Unfortunately, our analysis did not reach statistical significance – this could be partially attributed to the small sample size. Mönckeberg sclerosis is more common in the vasculature of the lower extremities; however, there is a growing body of evidence indicating that Mönckeberg sclerosis is not uncommon in the head and neck region (Tahmasbi-Arashlow *et al*. 2016, Omami 2017, Shahid *et al*. 2017, Frazier *et al*. 2018, Vijayan & Potluri 2020, Thomas *et al*. 2021). There are a few case reports in the literature that have indicated its presence in the thyroid (Micheletti *et al*. 2008, Tahmasbi-Arashlow *et al*. 2016).

The pathogenesis of MS is unknown; apoptotic and osteogenic processes involving osteoprotegerin and receptor activator of nuclear factor-κΒ ligand (RANKL) system have been proposed as potential pathogenetic factors (Schoppet *et al*. 2004). Medial arterial calcification associated with deficiency of CD73 (ACDC) is a rare monogenetic autosomal disease due to a loss of function mutation in the NT5E gene encoding the CD73 protein. A deficiency of CD73 affects adenosine metabolism that influences inorganic pyrophosphate and phosphate metabolism that ultimately creates a pro-calcification microenvironment. ACDC manifests as tortuous arteries with medial calcifications and hyperplasia, particularly in the lower extremities (Lanzer *et al*. 2014).

Mönckeberg MS has been overlooked as a rather benign condition; however, it is now recognized as a negative predictor of cardiovascular morbidity and mortality (Lanzer *et al*. 2014, Trimboli *et al*. 2021). Stiffening of the arterial wall in advanced MS results in decreased peripheral tissue perfusion and arterial flow stasis that can result in thrombus formation (Tahmasbi-Arashlow *et al*. 2016). It is commonly found in older patients with a medical history of hypertension, type II diabetes mellitus, and end-stage renal disease. In our cohort, there was no reported renal disease in either study group. The presence of MS was associated with dyslipidemia and hypertension, but not DMII. Whether the identification of MS can be used as a cardiovascular risk factor remains to be studied in prospective trials. The utilization of breast arterial calcifications visualized in mammography as a cardiovascular risk factor has recently been proposed (Trimboli *et al*. 2019, 2021).

Whether MS could also be implicated in cancer pathogenesis or disease progression has not been studied in clinical trials. The prevalence of MS in autopsy cases with cerebral infarction and advanced malignancy was significantly higher than in patients without malignant (Shichijo *et al*. 2021). In a recent study, Mönckeberg MS was associated with the presence of tumor budding, an adverse prognostic factor in invasive breast cancer (Kumarguru *et al*. 2020). In our study, patients with PTC and MS were more likely to have multifocal disease, invasion of the thyroid parenchyma, thyroid capsule, and extrathyroidal invasion but not lymph node disease.

The effect of MS on thyroid imaging was also evaluated. Indeed, the identification of calcifications in ultrasonography of the thyroid was increased in patients with MS. However, results of fine needle aspirations of the thyroid indicating malignancy or a non-diagnostic result were less common in cases. The presence of MS has been reported to mimic the malignant pattern of calcifications as seen in mammography (Saxena *et al*. 2005).

## Conclusion

Mönckeberg MS is an under-detected entity that could be found in the thyroid vessels of papillary thyroid cancer patients. This finding extrathyroidally has been associated with certain disease patterns and could be an indicator of cardiovascular risk. In our cohort, the presence of Mönckeberg sclerosis in the thyroid is not associated with differences in the metabolic profile and histopathologic characteristics of patients with differentiated thyroid carcinoma.

## Declaration of interest

The authors declare that there is no conflict of interest that could be perceived as prejudicing the impartiality of the study reported.

## Funding

This work did not receive any specific grant from any funding agency in the public, commercial, or not-for-profit sector.

## Ethics approval and consent to participate

This study was approved by the scientific board of our institution. Written informed consent for participation in this study was obtained from the patients prior to conducting this study.

## Availability of data and materials

The datasets used and/or analyzed during the current study are available from the corresponding author on reasonable request.

## Author contribution statement

VV, MB, and AC participated in data collection and writing of the manuscript. EZ and CZ contributed to the writing of the manuscript and abstract. KG completed the statistical analysis. PX contributed the pathology slides and the pathology data of the manuscript. AF, MS, and EA participated in the data collection process. All authors reviewed and approved the manuscript prior to submission.
